# Evaluation of the Robustness Under Alkanol Stress and Adaptability of Members of the New Genus *Halopseudomonas*

**DOI:** 10.3390/microorganisms12112116

**Published:** 2024-10-22

**Authors:** Simone Bertoldi, Pedro D. M. A. S. Mattos, Carla C. C. R. de Carvalho, Luzie Kruse, Stephan Thies, Hermann J. Heipieper, Christian Eberlein

**Affiliations:** 1 Department of Molecular Environmental Biotechnology, Helmholtz Centre for Environmental Research—UFZ, 04318 Leipzig, Germanypedro.santos.mattos@tecnico.ulisboa.pt (P.D.M.A.S.M.);; 2 Department of Bioengineering, iBB—Institute for Bioengineering and Biosciences, Instituto Superior Técnico, Universidade de Lisboa, 1049-001 Lisboa, Portugal; ccarvalho@tecnico.ulisboa.pt; 3 Institute of Molecular Enzyme Technology, Faculty of Mathematics and Natural Sciences, Heinrich Heine University Düsseldorf, 40204 Düsseldorf, Germany; 4 Institute of Bio- and Geosciences IBG-1: Biotechnology, Forschungszentrum Jülich, 52428 Jülich, Germany

**Keywords:** *Halopseudomonas*, *cis-trans* isomerase, solvent tolerance, *Pseudomonas*

## Abstract

Many species of the genus *Pseudomonas* are known to be highly tolerant to solvents and other environmental stressors. Based on phylogenomic and comparative genomic analyses, several *Pseudomonas* species were recently transferred to a new genus named *Halopseudomonas*. Because of their unique enzymatic machinery, these strains are being discussed as novel biocatalysts in biotechnology. In order to test their growth parameters and stress tolerance, five *Halopseudomonas* strains were assessed regarding their tolerance toward different *n*-alkanols (1-butanol, 1-hexanol, 1-octanol, 1-decanol), as well as to salt stress and elevated temperatures. The toxicity of the solvents was investigated by their effects on bacterial growth rates and presented as EC50 concentrations. Hereby, all *Halopseudomonas* strains showed EC50 values up to two-fold lower than those previously detected for *Pseudomonas putida*. In addition, the activity of the *cis-trans* isomerase of unsaturated fatty acids (Cti), which is an urgent stress response mechanism known to be present in all *Pseudomonas* species, was monitored in the five *Halopseudomonas* strains. Although several of the tested species were known to contain the *cti* gene, no significant phenotypic activity could be detected in the presence of the assayed stressors. A bioinformatic analysis of eight *cti*-carrying *Halopseudomonas* strains examining promotor binding sites, binding motifs and signal peptides showed that most of the *cti* genes have a lipoprotein signal peptide and promotor regions and binding motifs that do not coincide with those of *Pseudomonas*. These insights represent putative reasons for the absence of the expected Cti activity in *Halopseudomonas*, which in turn has always been observed in *cti*-carrying *Pseudomonas*. The lack of Cti activity under membrane stress conditions when the *cti* gene is present has never been documented, and this could represent potential negative implications on the utility of the genus *Halopseudomonas* for some biotechnological applications.

## 1. Introduction

Certain bacteria possess exceptional capabilities regarding tolerance and degradation potential toward organic compounds present in ecosystems impacted by anthropogenic organic pollutants or crude oil contamination [1,2]. These specialized bacteria play a crucial role in mitigating the harmful effects of such contaminants and promoting environmental remediation [3]. Whole toolboxes of different adaptive mechanisms enable them to thrive in contaminated environments and efficiently degrade complex organic compounds. By harnessing their enzymatic potential and metabolic pathways [4,5], these bacteria contribute to the restoration of ecosystems affected by organic pollutants, providing a promising avenue for addressing environmental challenges associated with these contaminations. Among all microorganisms pertinent to this matter, the well-studied genus *Pseudomonas* stands out as very broadly active among the Gram-negative proteobacteria [6].

Among *Pseudomonas* sp., solvent-tolerant strains are highly regarded as ideal biocatalysts for producing various chemicals in biotechnology, e.g., propionate or p-hydroxybenzoate [7,8]. However, in Germany, many of these strains, especially those of *P. putida* (excluding *P. putida* KT2440), are classified as biosafety level 2, imposing costly safety measures and reducing their attractiveness to the biotechnological industry. This classification is questioned because these bacteria have no pathogenic characteristics in their genome, and their safety is demonstrably guaranteed. In contrast, the U.S. FDA considers the *P. putida* group non-pathogenic [9]. The ongoing debate over the classification of *P. putida* has prompted suggestions of a new species, *P. alloputida*, which includes well-studied strains such as *P. putida* KT2440, *P. putida* S12 and *P. putida* DOT-T1E [10]. Apart from regulatory and taxonomic concerns, there is a crucial need to identify *Pseudomonas* or other bacterial strains with high tolerance to solvents and other stressors for establishing biotechnological production platforms [11,12].

In environments with toxic pollutants, fluctuating temperatures, or high levels of osmotic pressure, numerous species within the genus *Halopseudomonas* have been discovered that potentially exhibit tolerance to stress [13]. Previously classified within the *Pseudomonas* genus as part of the *Pseudomonas pertucinogena* lineage, these bacteria were recently reclassified as *Halopseudomonas* (also known as *Neopseudomonas*) within the *Pseudomonadaceae* family due to significant molecular and metabolic distinctions from other *Pseudomonas* species [14].

Genomic analyses have uncovered genes responsible for encoding various interesting enzymes in *Halopseudomonas*, including esterases and dehalogenases, as well as the potential for biosynthesis of polyhydroxyalkanoates (PHA), among others [13,15,16]. Additionally, there is speculation about the involvement of *Halopseudomonas* in breaking down human-made pollutants like oil or plastic waste [13,17,18,19,20]. In particular, in marine crude oil drilling sites, species like *H. aestusnigri* have been found to cope with alkanes and alkane alcohols. Despite these insights, information on the solvent tolerance of *Halopseudomonas* species has been limited. As they are potential candidates for the role of biotechnological workhorses [21], there is a necessity to elucidate their robustness against solvents. Testing their tolerance toward different *n*-alkanols (1-butanol, 1-hexanol, 1-octanol, 1-decanol) is suitable as a kind of benchmark test, and it has been applied already to novel bacterial strains, as well as genome-reduced strains, to compare them to their wildtype counterparts [22].

The purpose of this study is to evaluate the robustness of five members of the newly established *Halopseudomonas* genus against *n*-alkanols, heat shock and osmotic stress, and document their stress response. Therefore, the effective *n*-alkanol concentration for reducing cell growth by 50% compared to the control (EC50) was determined for five *Halopseudomonas* strains. Among the five mentioned strains, three carry the gene for the *cis-trans* isomerase (Cti) of membrane fatty acids, with its activity being a unique urgent stress response mechanism for stabilizing the bacterial membrane and a potential taxonomic marker for the genus *Pseudomonas* [11,23]. Only very few bacterial genera carry the gene for the periplasmic Cti, a cytochrome c-type protein with a heme-binding motif [23,24]. Given that the gene is constitutively transcribed in *Pseudomonas* spp., it was suggested that the protein remains in the periplasmic region until the fluidity of the membrane requires it to move toward its *cis*-configurated unsaturated fatty acid substrates [24]. After its action, the membrane is reduced in fluidity and the Cti returns to its original periplasmic location. The assessment of Cti activity in the recent genera *Halopseudomonas* is still pending. Therefore, the role of this enzyme in the robustness of the strains, which has yet to be determined, was investigated in a total of eight *Halopseudomonas* strains bioinformatically. Of these, three selected strains were also tested for a Cti phenotype upon exposure to *n*-alkanols.

## 2. Materials and Methods

### 2.1. Bacterial Strains and Media

Unless otherwise indicated, a modified Hartmans [25] mineral salt medium was used to grow the cultures, usually with 18 mM sebacic acid (decanedioic acid). The medium was prepared with the following solutions: 

Mineral Salts Solution (100×)—In 1 L of distilled water, the following salts were added: 100 g of NH_4_Cl, 70 g of Na_2_HPO_4_ × 2H_2_O, 30 g of NaCl and 28 g of KH_2_PO_4_.

Trace Elements Solution (500×)—In 1 L of distilled water, the following compounds were added: 73 mL of HCl (37%), 50 g of MgSO_4_ × 7H_2_O, 5 g of FeSO_4_ × 7H_2_O, 5 g of EDTA, 3.25 g of H_3_BO_3_, 3.2 g of ZnCl_2_, 2.5 g of MnSO_4_ × H_2_O, 0.5 g of CaCl_2_ × 6H_2_O, 0.3 g of BaCl_2_, 0.18 g of CoSO_4_ × 7H_2_O and 0.18 g of CuSO_4_ × 5H_2_O.

The rifampicin-resistant *Halopseudomonas* strains (*H. aestusnigri* VGXO14R, *H. oceani* KX20R, *H. litoralis* 2SM5R and *H. bauzanensis* BZ93R) were grown in a modified Hartmans mineral medium (0.3% NaCl) with 18 mM sebacic acid (decanedioic acid) as a carbon source at 30 °C and 150 rpm at a pH of 6.9 [21]. A spontaneous rifampicin-resistant mutant of *H. pachastrellae* was isolated as follows: *H. pachastrellae* was streak-plated on an LB-Agar plate (5 g/L yeast extract, 10 g/L tryptone, 10 g/L glucose and 20 g/L agar) and incubated at 30 °C for 48 h. The colonies formed were then streak-plated on another 25 mL LB-Agar plate with 25 μg/mL of the rifampicin. After 48 h, this process was repeated. Once the final colonies were formed, the bacterium was grown under the conditions previously described [21]. The day before each experiment, 50 mL overnight cultures (around 15 h) were prepared in 500 mL DURAN wide neck Erlenmeyer flasks containing the aforementioned modified Hartmans mineral medium, achieving a final optical density (OD_560_) of 2–3. For the subsequent experiments, a 50 mL culture was grown at an OD_560_ of 0.1–0.2 in 250 mL screw cap glass bottles (30 °C, 150 rpm, pH of 6.9). The growing conditions were the same as for the overnight culture. The bottles were kept in an orbital shaking water bath (GFL 1092) throughout the experiment (30 °C and 180 rpm). 

For each experiment, the medium was prepared in bulk and distributed in each of the 250 mL screw cap glass bottles. Depending on the achieved overnight OD, the amount of inoculum was chosen to make up an initial OD of 0.1–0.2. Rifampicin solution was added to each bottle to reach a final concentration of 25 μg/mL. The rifampicin solution was always protected from the light with tin foil and stored at −20 °C. Sebacic acid (≥99%), rifampicin (≥97%), dimethyl sulfoxide (DMSO, ≥99.9%) as well as all applied *n*-alkanols, NaCl and acetone were obtained from Merck (Darmstadt, Germany). 

In addition, *Pseudomonas taiwanensis* VLB120 was used as a benchmark for solvent tolerance and as a positive control for Cti activity. The cultivation followed a standard procedure using mineral salt medium [25] and disodium succinate (24.7 mM) as a carbon and energy source at 30 °C and 150 rpm [22].

The monitoring of bacterial growth was carried out by measuring the optical density at 560 nm in an Agilent Cary 100 UV–Vis Spectrophotometer (Waldbronn, Germany) every hour. The release of a red pigment was observed to be absorbed at the same wavelength. In an attempt to reduce its effect, a 1:10 dilution with a 50 mM phosphate buffer (phosphate buffer blank) was made in a single-use polystyrene semi-micro cuvette. This reduced the contribution of the released red pigment to the real OD.

### 2.2. Growth Under Solvent, Osmotic and Temperature Stress

The bacterial stress experiments were conducted with 1M NaCl (osmotic stress); 45 °C (heat shock); 0–100 mM 1-butanol; 0–7 mM 1-hexanol; 0–0.7 mM 1-octanol; and 0–0.25 mM 1-decanol (solvent stress) under the aforementioned growth conditions (modified Hartmans mineral medium with 18 mM sebacic acid at 30 °C and 150 rpm, pH of 6.9). The concentrations of the *n*-alkanols mentioned were chosen according to their log P_octanol/water_ (partitioning coefficient between octanol and water [26]). The stressed bacterial cultures were compared to a control without the stressor. The stressors were added and exposed, respectively, to exponentially growing bacteria. After 3 h in the presence of the stressors, the cultures were centrifuged at 10,000× *g* for 15 min in a Hermle Z 383 K table cooling centrifuge. The pellets were then resuspended in 1.75 mL of the aforementioned phosphate buffer and centrifuged at 14,000× *g* for 7 min, using 2 mL Eppendorf tubes. The buffer was then removed, and the pellets were frozen at −20 °C to store them for the later extraction of membrane lipids. Growth inhibition caused by the toxic compounds was measured by comparing the percentage difference in the growth rates between intoxicated cultures with that of the control [27]. Growth rates in the first three hours after stressor addition and growth inhibition were calculated as published earlier [22].

### 2.3. Extraction of Membrane Lipids

Lipid extraction was conducted according to Bligh and Dyer [28]. Using the method of Morrison, Smith [29], fatty acid methyl esters (FAMEs) were synthesized by 15 min incubation at 95 °C in 0.6 mL of boron trifluoride–methanol complex (20% MeOH; Sigma Aldrich, Munich, Germany). FAME analysis was performed using gas chromatography with a flame ionization detector (GC-FID, Agilent Technologies, Waldbronn, Germany 6890N Network GC System, 7683B Series Injector). The instrument used a CP-Sil 88 column (Varian CP7488, Palo Alto, CA, USA) in the stationary phase and helium as a carrier gas. The peak areas of the FAMEs were used to determine their relative amounts. The fatty acids were identified by a co-injection of authentic reference compounds obtained from Supelco (Bellefonte, PA, USA). Equation 1 was used to calculate the degree of saturation (DoS) of the FAMEs. It is the quotient of the sum of the saturated FAMEs with the sum of the unsaturated FAMEs.

Degree of Saturation = (% C16:0 + % C18:0)/sum of unsaturated fatty acids (1)

The *trans/cis* ratio was calculated by taking the sum of the FAME of palmitoleic acid C16:1 ∆^9^*cis*) and *cis*-vaccenic acid (C18:1 ∆^11^*cis*) as the divisor and the sum of their corresponding trans configuration as the dividend (Equation (2)).

*trans/cis* ratio = (% C16:1∆^9^*trans* + % *C*18:1∆^11^*trans*)/(% C16:1∆^9^*cis* + % *C*18:1∆^11^*cis*) (2)

### 2.4. Bioinformatic Analysis

The *cti* genes were identified by BLAST analysis, using the *cti* sequence of *Pseudomonas taiwanensis* VLB120 as a query to search the genomes of *Halopseudomonas* species. The binding motifs conservation (HMM logo) was studied. The derived protein sequences of the following strains were aligned and analyzed: *H. aestusnigri* VGXO14R, *H. oceani* KX20R and *H. pachastrellae* JCM 12285, *P. abyssi* MT5, *H. gallaeciensis* V113, *H. pelagia* CL-AP6, *H. sabulinigri* JCM 13963 and *H*. sp. RR6. The prediction of the signal peptides was performed with the use of the online tool SignalP 6.0 described by Nielsen and colleagues [30]. It has to be mentioned that *P. abyssi* MT5 was also proposed to be a member of the genus *Halopseudomonas*. However, due to the lack of an available strain type in culture collections, this could not be implemented yet [14].

## 3. Results and Discussion

### 3.1. Growth of Five Halopseudomonas Strains in Mineral Medium

The observed reduction in growth rates under stress conditions in comparison to unchallenged cells is an established parameter to quantify the impact of a stressor [22]. As a benchmark, the growth rates of all five tested *Halopseudomonas* strains on sebacic acid in mineral media under the applied cultivation conditions were determined. Among the five species, the growth rates were within the same order of magnitude and comparable to previously published results [21]. *H. aestusnigri* and *H. oceani* achieved the highest growth rates among the cultivated strains (Table 1).

### 3.2. Distribution of Cti Genes Among Halopseudomonas 

Having been classified as Pseudomonadales and until recently even belonging to the genus *Pseudomonas*, the genomes of *Halopseudomonas* species were searched for genes encoding homologs of fatty acid *cis/trans* isomerase. This enzyme is characteristic among *Pseudomonas* species, and it is a key trait of their robustness against hydrophobic chemicals, being an urgent response mechanism against membrane destabilization [23]. Notably, putative *cti* genes were present in some investigated *Halopseudomonas* species but are apparently not a shared feature of the genus *Halopseudomonas* (Table 2) The *Halopseudomonas* strains carrying a *cti* gene are listed in Table 2. Since a gene for proteins homologous to the Cti of *P. taiwanensis* VLB120 was found in the genomes of *H. aestusnigri*, *H. oceani* and *H. pachastrellae*, Cti activity was determined in five different *Halopseudomonas* species (Table 2) based on the incubation at elevated temperatures, and osmotic and solvent stress (1-butanol, 1-hexanol, 1-octanol, 1-decanol). 

### 3.3. Growth Under Solvent, Osmotic and Temperature Stress

**Figure 1:**
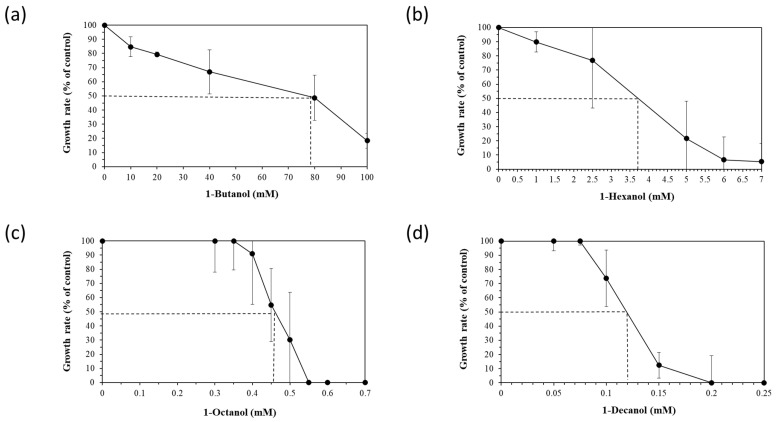
Growth rates (black circles) and *trans/cis* ratios (white diamonds) of *H. aestusnigri* incubated with different *n*-alkanols: (**a**) 1-butanol, (**b**) 1-hexanol, (**c**) 1-octanol and (**d**) 1-decanol. The EC50 (effective concentration for reducing cell growth by 50% compared to the control) is represented by a dotted line. *n* = 3.

To assess the robustness of the five selected *Halopseudomonas* strains against a chemical stressor, the effect of different concentrations of four *n*-alkanols (1-butanol, 1-hexanol, 1-octanol and 1-decanol), added during the early exponential (logarithmic) growth phase, was tested. The exponential growth phase is usually chosen for stressor addition to provide a controlled and consistent environment to study the immediate and specific responses of actively dividing cells that do not face nutrient limitations yet. Relative growth inhibition was calculated between the growth rates of the control and stressed cultures and is showcased for *H. aestusnigri* in Figure 1. The EC50 (effective concentration for reducing cell growth by 50% compared to the control) was deduced from the growth inhibition for each *n*-alkanol (dotted line in Figure 1).

The approximate EC50 values for all strains tested are given in Table 3. A difference between the *cti* carriers (*H. aestusnigri*, *H. pachastrellae* and *H. oceani*) and the non-*cti*-carriers (*H. bauzanensis* and *H. litoralis*) can be observed, because the latter have comparatively lower EC50 values. However, there are exceptions to this trend, such as *H. litoralis* exposed to 1-butanol and 1-decanol, which showed similar EC50 values compared to the *cti* carriers. Simultaneously, *H. oceani* exhibits a lower tolerance toward 1-butanol and 1-octanol, but a higher tolerance toward 1-decanol. The EC50 values of the *Halopseudomonas* strains were at least half compared to those of *P. taiwanensis* for every *n*-alkanol except for 1-hexanol (Table 3).

**Figure 2:**
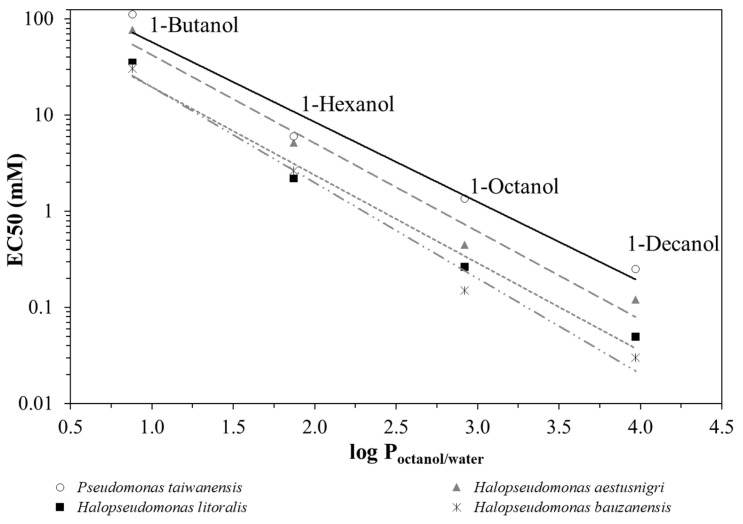
Correlation between EC50 and log P_octanol/water_ values for 1-butanol, 1-hexanol, 1-octanol and 1-decanol. As examples, *H*. *bauzanensis* (mix of dashed and dotted grey trendline), *H. aestusnigri* (long dashed grey trendline), *H*. *litoralis* (short dashed grey trendline) and *P. taiwanensis* VLB120 (solid black trendline) are shown.

By plotting the EC50 values as a function of the partitioning coefficient between octanol and water, log P_octanol/water_ [26] of the respective alkanol (Figure 2), it can be seen that *P. taiwanensis* VLB120 outperforms the *Halopseudomonas* strains regarding its robustness against *n*-alkanols of increasing hydrophobicity. *H. aestusnigri* can be considered the most tolerant among the strains tested. Typically, substances with a log P_octanol/water_ between 1 and 4, such as long-chain alkanols, aromatics and esters, exhibit toxicity to microorganisms even at very low concentrations because they tend to accumulate in the cytoplasmic membrane [36,37,38].

After the toxicity tests, the membrane fatty acids were extracted to calculate the activity of the Cti. The phospholipid fatty acid profile of the strains showed the presence of the following fatty acids: C16:0, C16:1*trans*, C16:1*cis*, 17*cyclo*, C18:0, C18:1*trans* and C18:1*cis*. In accordance with their percentage distribution, the degree of saturation and the *trans/cis* ratio of each species’ control were calculated (Table 4). The *trans/cis* ratio in the controls without solvent was either zero or very close to zero for all *Halopseudomonas* strains. Remarkably, despite the incubation with four *n*-alkanols, elevated temperature, or osmotic stress, the *trans/cis* ratio remained stable in the *Halopseudomonas* strains, whereas a clear increase occurred in strain VLB120. Maximum *trans/cis* ratios are shown in Table 4. The *trans/cis* ratios of *H. litoralis* and *H. bauzanensis*, both of which do not have a *cti* homolog, were also measured routinely, and the values were always close to zero, regardless of the presence or absence of a solvent.

The results from Table 4 show that the Cti had neglectable or no activity upon solvent exposure in all tested *Halopseudomonas* strains. This was made clear through the comparison with *P. taiwanensis* VLB120, which was used as positive control. Strains *H. litoralis* and *H. bauzanensis* did not show any Cti activity, which was expected due to the *cti* gene’s absence. Increased *trans/cis* ratios of more than 1 upon exposure to a stressor are documented for various *cti*-carrying bacteria, like for *Vibrio cholerae* under starvation stress [39], as well as for *Pseudomonas putida* S12 [27], and for *Alcanivorax borkumensis* SK2 under solvent stress [40]. Also, for *Methylococcus capsulatus* Bath, a small increase in *trans/cis* ratios was found upon solvent exposure [41]. Hence, we tested other stressors known to trigger *cis/trans* isomerization [42]. However, Cti activity was observed neither under osmotic nor heat stress conditions (Table 4). Nevertheless, an extremely low or non-existent increase in *trans/cis* ratios like those shown by the *Halopseudomonas* strains investigated here had never been observed before. In fact, such a discrepancy between a Cti genotype and phenotype had never been described, to our knowledge. This discrepancy is also consistent with the fact that all tested strains of the genus *Halopseudomonas* were significantly less solvent-tolerant than all strains of the closely related genus *Pseudomonas*. However, this leaves open the question of why *Halopseudomonas* strains do not show Cti activity even though they possess the corresponding *cti* gene.

### 3.4. Binding Motifs Comparison and Signal Peptides

In light of these experimental results, we tried to find mutual differences between *Halopseudomonas* Cti and Cti with proven activity from *Pseudomonas* spp. Therefore, we included all of the Cti identifiable in the *Halopseudomonas* clade, thereby adding another five strains (*P. abyssi* MT5, *H. gallaeciensis* V113, *H. pelagia* CL-AP6, *H. sabulinigri* JCM 13963 and *H.* sp. RR6) to the *Halopseudomonas* strains that were assessed phenotypically. Using the Multiple Sequence Alignment tool from Uniprot and a hidden Markov model, the similarities in common amino acid sequences between the tested *Halopseudomonas* strains become apparent, as well as their differences compared to the *Pseudomonas* strains [43] (Figure S1).

Even though the Cti proteins in all strains investigated so far do not have a high degree of sequence identity, their mode of action is the same. Hence, a heme binding motif should be present, which will allocate the ferrous cation, as described previously [24,43,44]. In addition, three putative phosphorylation sites (PGSTEAL, SRTPSG and DMNRYENL) are expected. Within the known Ctis of *Pseudomonas*, there is a certain sequence variation. By comparing the sequences with the reference Cti of *P. aeruginosa* PAO1, *P. capeferrum* TDA1 [45] shows 65.32% sequence identity, and *H. aestusnigri* and *H. oceani* as well as *H. pachastrellae* show around 49% sequence identity (query coverage for all between 97 and 100%, and E value for all 0.00) (Figure S1, Supplementary Materials). For Pseudomonads, this was also shown in the work of Mauger and colleagues, but the mentioned motifs, however, were found to be conserved [43]. The heme binding site and the proline residue are conserved in the *Halopseudomonas* strains as well. However, there is an observable difference in the *Halopseudomonas* strains compared to the *Pseudomonas* strains at the putative phosphorylation sites, more prominently at the expected PGSTEAL and DMNRYENL sites (conserved as XGREQL and DINRYQNL, respectively). The differences in the phosphorylation sites could be an explanation for Cti inactivity. Support for the hypothesis of Cti inactivity because of the non-conserved phosphorylation sites comes from another study with the *cti*-carrying *Methylococcus capsulatus* Bath [41], whose diminished Cti activity could be related to the different phosphorylation sites (SSPKERL, SRTPPG, DLSRTDNP). However, there may be other factors influencing this matter. For instance, as mentioned in the introduction, the Cti is a periplasmic protein, hence the need for a signal peptide that will allow the cell to recognize the protein’s translocation paths.

In order to direct the Cti to its final cellular compartment, namely the periplasm, a signal peptide is required, which is usually a short peptide sequence, typically 15–30 amino acids long and often located at the N-terminus. Therefore, the signal peptides of the Ctis were compared using the online software SignalP 6.0 [30]. It predicts the presence of signal peptides and the location of their cleavage sites. Table 5 gives an overview of the results. Figure S2 (Supplementary Materials) shows the software’s output for the Cti of *H. aestusnigri*, but it is representative of six out of eight *Halopseudomonas* strains (except for *H. oceani* and *H. pelagia*), and it detected the presence of a Sec-dependent lipoprotein signal peptide cleaved by the signal peptidase II (SPase II) [46], resulting in a membrane-anchored periplasmic Cti. In contrast to this, in the putative protein of the *cti* gene in *H. oceani*, *P. capeferrum* TDA1 and *P. aeruginosa* PAO1, a Sec-dependent signal peptide cleaved by the signal peptidase I (SPase I) is present (Table 5). Hence, all *Halopseudomonas* strains bear a signal peptide in their potential Cti sequence, but only in *H. oceani* and *H. pelagia* is the Cti not anchored in the membrane after membrane translocation. Hence, constrained flexibility of the enzyme by n-terminal covalent attachment to the membrane could be a reason for the very low or absent activity of the Cti in *Halopseudomonas* strains, but not in *H. oceani* and *H. pelagia*. Nevertheless, enzymes such as thioesterases or peptidases might also hydrolyze the bond between the Cti and the membrane and thereby release a membrane-anchored Cti.

## 4. Conclusions

Five strains of the barely explored genus *Halopseudomonas* were subjected to a series of toxicity tests in the presence of four *n*-alkanols. A rapid decrease in OD_560_ was consistently observed upon incubation with the *n*-alkanols tested. The EC50 values for each *n*-alkanol were considerably lower than those observed with the selected tolerant *Pseudomonas* strains.

Remarkably, the *cis-trans* isomerase of unsaturated fatty acids, the activity of which is considered a typical immediate response trait of Pseudomonads leading to a fast rigidification of the membrane, appears not be conserved among *Halopseudomonas* strains, but encoded only in the genomes of some species. However, even the latter strains were not able to elevate their *trans/cis* ratio in response to alkanols, or to other stressors including salt and elevated temperature. This is the first study showing a discrepancy between a *cti* genotype and missing Cti activity in the phenotype. It is hypothesized that a membrane-anchored Cti and non-functional phosphorylation sites could be responsible for this. The lower membrane stress tolerance compared to other *Pseudomonas* might have negative implications on the utility of the genus *Halopseudomonas* as whole-cell biocatalysts in certain biotechnological applications besides the described utilization of aliphatic plastics [15,19]. The insights first gained here into the physiology of *Halopseudomonas* support a niche adoption of these species compared to their robust versatile relatives among the Pseudomonadaceae clade.

## Figures and Tables

**Table 1:** Growth rates of the five tested *Halopseudomonas* strains on sebacic acid in mineral media evaluated during the first five hours of growth; *n* = 2 for *H. oceani* [31], *H. bauzanensis* [32] and *H. aestusnigri* [33], *n* ≥ 1 for *H. pachastrellae* [34] and *H. litoralis* [35].

Organism	Growth Rate µ [h^−1^]	Standard Deviation
*H. aestusnigri* VGXO14R	0.47	±0.016
*H. litoralis* 2SM5R	0.29	±0.005
*H. oceani* KX20R	0.32	±0.018
*H. bauzanensis* BZ93R	0.27	±0.031
*H. pachastrellae* JCM 12285	0.22	n.d.

**Table 2:** Overview of the strains used in the study for bioinformatic analyses, including the five analyzed for tolerance toward *n*-alkanols. *P. abyssi* MT5 was also proposed to be a member of the genus *Halopseudomonas*. However, due to the lack of an available strain type in culture collections, this is still to be implemented [13].

Organism	Accession Number	Cti-Gene Present	Cti Accession Number	Stress Tolerance Tested
*H. litoralis* 2SM5R	NZ_LT629748	-	-	+
*H. bauzanensis* BZ93R	NZ_FOGN01000016	-	-	+
*H. aestusnigri* VGXO14R	NZ_NBYK01000004	+	WP_088275311	+
*H. oceani* KX20R	NZ_PPSK01000004	+	WP_229744351	+
*H. pachastrellae* JCM 12285	NZ_FOUD01000015	+	WP_083727452	+
*P. abyssi* MT5	NZ_NTMR00000000	+	WP_096004757	-
*H. gallaeciensis* V113	NZ_LMAZ00000000	+	WP_118129608	-
*H. pelagia* CL-AP6	NZ_AROI00000000	+	WP_235801961	-
*H. sabulinigri* JCM 13963	NZ_LT629763	+	WP_092284319	-
*Halopseudomonas* sp. RR6	NZ_CP079801	+	WP_238871345	-

**Table 3:** EC50 values expressed in mM of each strain toward the four *n*-alkanols tested. C4 = 1-Butanol; C6 = 1-Hexanol; C8 = 1-Octanol; C10 = 1-Decanol. For *H. pachastrellae*, *H. oceani* and *H. litoralis*, *n* ≥ 1. For *H. aestusnigri*, *H. litoralis* and *P. taiwanensis*, *n* = 3.

Organism	C4	C6	C8	C10
*Pseudomonas taiwanensis* VBL120	111.3	6.0	1.4	0.3
*Halopseudomonas aestusnigri* VGXO14R	77.0	3.8	0.5	0.1
*Halopseudomonas litoralis* 2SM5R	35.0	2.2	0.3	0.1
*Halopseudomonas oceani* KX20R	45.9	5.1	0.3	0.2
*Halopseudomonas bauzanensis* BZ93R	30.4	2.7	0.2	0.0
*Halopseudomonas pachastrellae* JCM 12285	73.8	9.5	0.5	0.1

**Table 4:** Maximum values of *trans/cis* ratios of *P. taiwanensis*, *H. aestusnigri*, *H. litoralis*, *H. oceani* and *H. pachastrellae* cells under osmotic stress (1 M NaCl), temperature stress (45 °C) and upon exposure to the four *n*-alkanols tested. C4 = 1-butanol (0–100 mM); C6 = 1-hexanol (0–7 mM); C8 = 1-octanol (0–0.7 mM); C10 = 1-decanol (0–0.25 mM); control = without stressor. *n* ≥ 2.

Strain	Control	C4	C6	C8	C10	1 M NaCl	45 °C
*Pseudomonas* *taiwanensis* VBL120	0.39	0.72	1.16	1.5	1.49	n.d.	n.d.
*Halopseudomonas aestusnigri* VGXO14R	0.05	0.02	0.04	0.02	0.06	0.08	0.02
*Halopseudomonas litoralis* 2SM5R	0.00	0.00	0.00	0.00	0.00	0.00	0.00
*Halopseudomonas oceani* KX20R	0.001	0.01	0.01	0.00	0.03	0.00	0.01
*Halopseudomonas bauzanensis* BZ93R	0.02	0.01	0.02	0.01	0.04	0.13	0.00

**Table 5:** *Halopseudomonas* strains’ Cti location prediction using the SignalP 6.0 software. The signal peptide is predicted to either be Sec-dependent and cleaved by the signal peptidase I (Sec/SPI) or a lipoprotein signal peptide and cleaved by the signal peptidase II (Sec/SPII).

Organism	Output SignalP 6.0	Probability	Assumed Protein Location
*P. aeruginosa* PAO1	Sec/SPI	0.9992	Periplasmic
*P. capeferrum* TDA1	Sec/SPI	0.9991	Periplasmic
*H. aestusnigri* VGXO14R	Sec/SPII	0.7513	Periplasmic and membrane-anchored
*H. oceani* KX20R	Sec/SPI	0.9987	Periplasmic
*H. pachastrellae* JCM 12285	Sec/SPII	0.9996	Periplasmic and membrane-anchored
*P. abyssi* MT5	Sec/SPII	0.9996	Periplasmic and membrane-anchored
*H. gallaeciensis* V113	Sec/SPII	0.9989	Periplasmic and membrane-anchored
*H. pelagia* CL-AP6	Sec/SPI	0.9952	Periplasmic
*H. sabulinigri* JCM 13963	Sec/SPII	0.9997	Periplasmic and membrane-anchored
*Halopseudomonas* sp. RR6	Sec/SPII	0.9962	Periplasmic and membrane-anchored

## Data Availability

The original contributions presented in the study are partially included in the article/Supplementary Material, and further inquiries can be directed to the corresponding author.

## Supplementary Materials

The following supporting information can be downloaded at: https://www.mdpi.com/article/10.3390/microorganisms12112116/s1, Figure S1: Multiple Sequence Alignment of the *cis-trans* isomerase amino acid sequences belonging to *H. pelagia*, *H. sabulinigri*, *Halospeudomonas* sp. RR6, *H. pachastrellae*, *P. abyssi*, *H. gallaeciensis*, *H. aestusnigri* and *H. oceani.* Figure S2: *H. aestusnigri* SignalP 6.0 signal peptide prediction output (representative of all mentioned *Halopseudomonas* strains excluding *H. oceani*). The first 70 amino acids are shown (x-axis) and plotted against the signal peptide’s probability. The dark green dotted line is the cleavage site (CS). To the right of the CS, the software does not find a signal peptide, thus it predicts “OTHER”, which means it is the protein itself. A blue line indicates the presence of a characteristic cysteine residue, signalizing the presence of a Sec-dependent lipoprotein signal peptide. To the left of the CS, the lipoprotein signal peptide is predicted. It is divided into two lines (red and orange), which correspond to two constitutive regions of the signal peptide (N and H, respectively). The signal peptide is predicted to be Sec-dependent and cleaved by the signal peptidase II (SPase II), as indicated by the legend in the upper right corner.
