# Supplementary material for: Evaluation of the Robustness Under Alkanol Stress and Adaptability of Members of the New Genus Halopseudomonas
Source: Microorganisms. 2024 Oct 22;12(11):2116. doi: 10.3390/microorganisms12112116 (PMC11596728; doi:10.3390/microorganisms12112116)
Supplement: Supplementary file 1 [file microorganisms-12-02116-s001.zip › microorganisms-3231435-supplementary.pdf]

Evaluation of the robustness toward alkanol stress and adaptability of members of the new genus *Halopseudomonas*

Simone Bertoldi[1], Pedro D.M.A.S. Mattos[1], Carla C.C.R. de Carvalho[2], Luzie Kruse[3], Stephan Thies[4], Hermann J. Heipieper[1], Christian Eberlein[1]

[1]Department of Molecular Environmental Biotechnology, Helmholtz Centre for Environmental Research - UFZ, Leipzig, Germany
[2]iBB- Institute for Bioengineering and Biosciences, Department of Bioengineering, Instituto Superior Técnico, Universidade de Lisboa, Lisboa, Portugal
[3]Institute of Molecular Enzyme Technology, Heinrich Heine University Düsseldorf, Faculty of Mathematics and Natural Sciences, Düsseldorf, Germany
[4]Institute of Bio- and Geosciences IBG-1: Biotechnology, Forschungszentrum Jülich, Jülich, Germany

Supplementary material

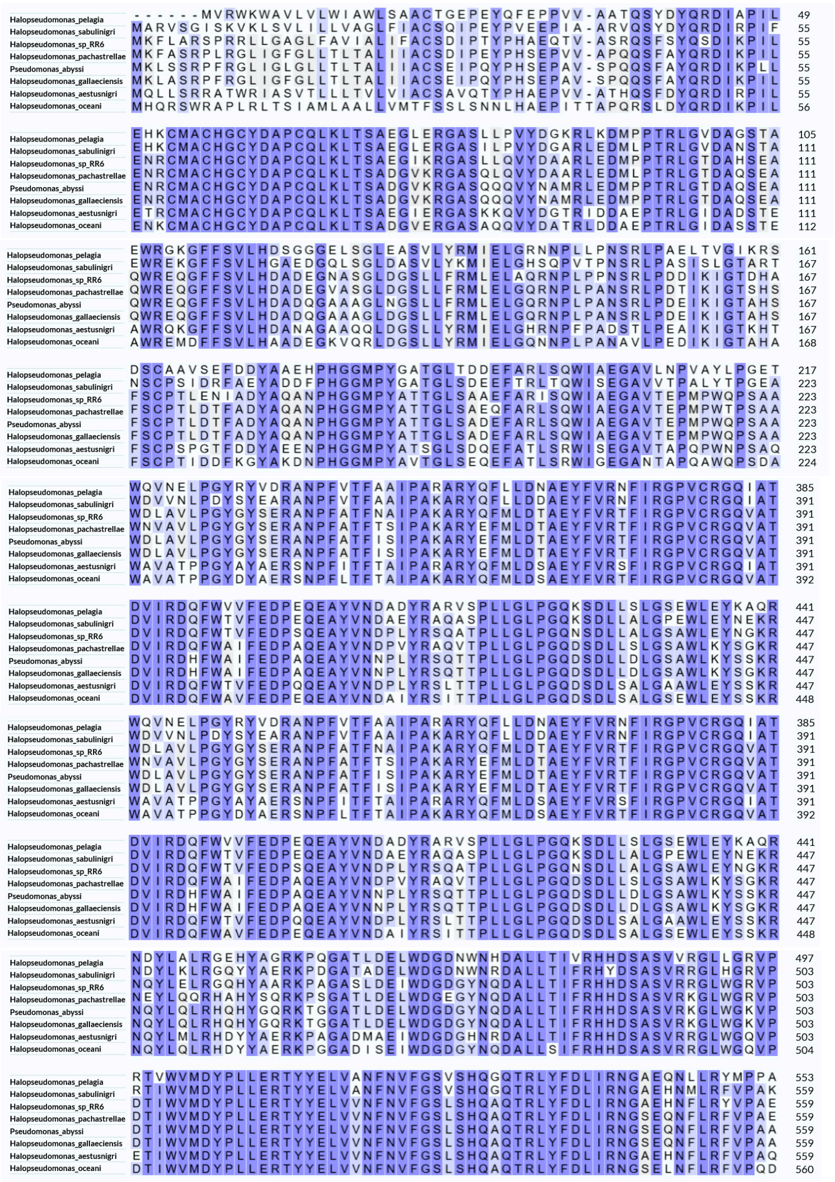

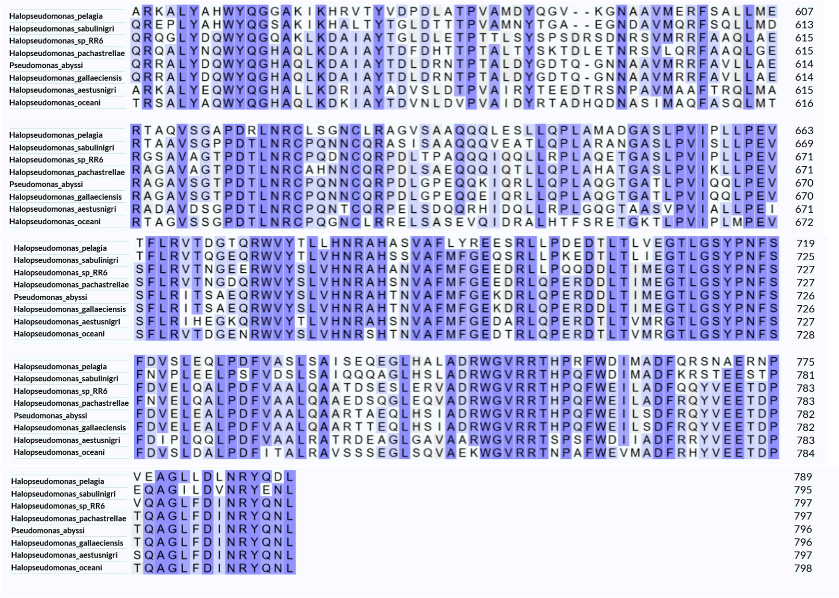

Figure S1: Multiple Sequence Alignment of the *cis-trans* isomerase amino acid sequences belonging to *H. pelagia, H. sabulinigri, Halospeudomonas* sp. RR6, *H. pachastrellae, P. abyssi, H. gallaeciensis, H. aestusnigri* and *H. oceani*.

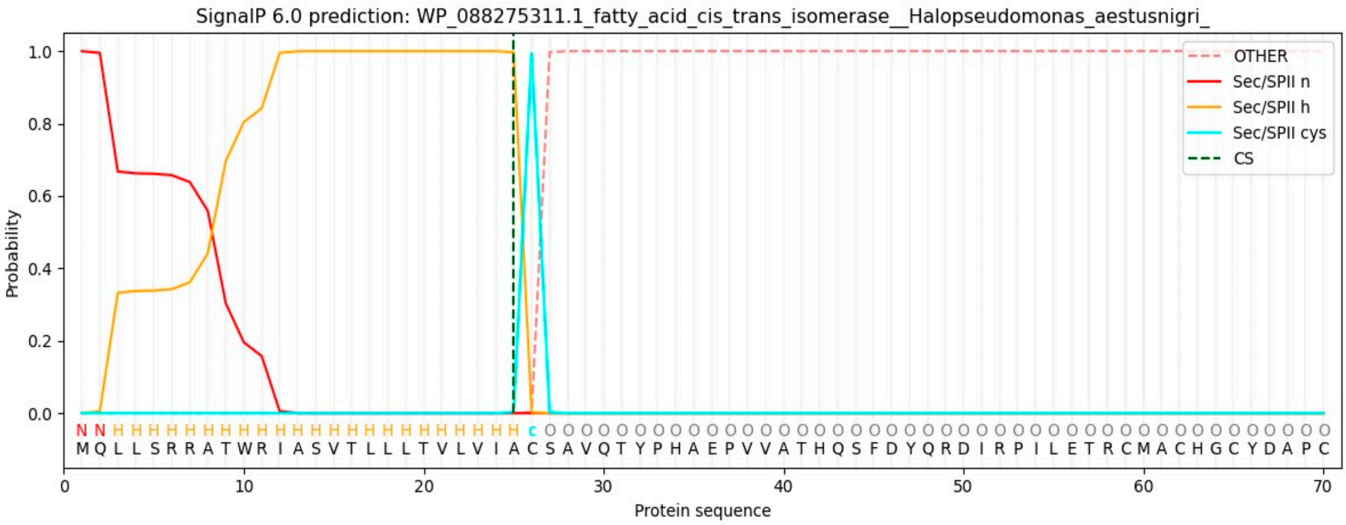

Figure S2: *H. aestusnigri* SignalP 6.0 signal peptide prediction output (representative of all mentioned *Halopseudomonas* strains excluding *H. oceani*). The first 70 amino acids are shown (x-axis) and plotted against the signal peptide's probability. The dark green dotted line is the cleavage site (CS). To the right of the CS, the software does not find a signal peptide, thus it predicts "OTHER", which means it is the protein itself. A blue line indicates the presence of a characteristic cysteine residue, signalizing the presence of a Sec-dependent lipoprotein signal peptide. To the left of the CS, the lipoprotein signal peptide is predicted. It is divided into two lines (red and orange), which correspond to two constitutive regions of the signal peptide (N and H, respectively). The signal peptide is predicted to be Sec-dependent and cleaved by the signal peptidase II (SPase II), as indicated by the legend in the upper right corner.
